# Literary Notes

**Published:** 1908-02

**Authors:** 


### LITERARY NOTES.

The *Columbus Medical Journel,* published in the capitol city of Ohio, loses from its editorial chair Dr. James U. Barnhill, who has conducted the journal for the past nine years. His successor will be fortunate if he continues for the journal the same ethical status and importance that it maintained under Dr. Barnhill's administration.

The *Fort Wayne Medical Journal-Magazine,* which has been in existence for twenty-five years, ceased publication with its December, 1907, issue. We shall miss the Journal-Magazine from our exchange files as we had learned to regard it as one of the best of its class. Its corps of editors were sterling men, who enjoyed the confidence and respect of their confreres.

The Journal of the Indiana State Medical Association has made its appearance as a handsome quarto, well dressed and looks as if it might be equal to the responsibilities it assumes. The editor is Dr. Albert E. Bulson and the assistant editor is Dr. Benjamin P. Weaver. The journal is published at Fort Wayne.

The *Albany Medical Annals* in its issue for January, 1908, publishes thirteen articles by the officers of the Bender Hygienic Laboratory, comprising one hundred and sixty pages. Some of the articles are illustrated and all are of scientific value. It is called the Bender Laboratory number.

The National Hospital Record, heretofore a standard octavo monthly, has changed to a twice-a-month journal, and, also, has changed its form to,—what shall we call it,—a royal imperial octavo, perhaps; at all events it is too ill-shapen for filing or binding. It is a pity such a useful publication should appear in such an unwieldy form.

